# Distinct Motivational Effects of Contingent and Noncontingent Rewards

**DOI:** 10.1177/0956797617693326

**Published:** 2017-05-10

**Authors:** Sanjay G. Manohar, Rebecca Dawn Finzi, Daniel Drew, Masud Husain

**Affiliations:** 1 Nuffield Department of Clinical Neurosciences, University of Oxford; 2 Department of Psychology, University of California San Diego

**Keywords:** motivation, rewards, eye movements

## Abstract

When rewards are available, people expend more energy, increasing their motivational vigor. In theory, incentives might drive behavior for two distinct reasons: First, they increase expected reward; second, they increase the difference in subjective value between successful and unsuccessful performance, which increases contingency—the degree to which action determines outcome. Previous studies of motivational vigor have never compared these directly. Here, we indexed motivational vigor by measuring the speed of eye movements toward a target after participants heard a cue indicating how outcomes would be determined. Eye movements were faster when the cue indicated that monetary rewards would be contingent on performance than when the cue indicated that rewards would be random. But even when the cue indicated that a reward was guaranteed regardless of speed, movement was still faster than when no reward was available. Motivation by contingent and certain rewards was uncorrelated across individuals, which suggests that there are two separable, independent components of motivation. Contingent motivation generated autonomic arousal, and unlike noncontingent motivation, was effective with penalties as well as rewards.

When rewards are available, people are willing to expend more energy. The generalized drive by which rewards energize action is known as *motivational vigor* (Berridge, 2004; Niv, Joel, & Dayan, 2006). In theory, incentives might drive behavior for two closely related—but distinct—reasons: First, they increase *expected reward*; second, they increase the value difference between successful and unsuccessful performance, which thus introduces greater *contingency*. In previous studies of motivation, these two aspects of motivational vigor—stimulated by noncontingent versus contingent incentives—have never been directly compared.

Contingency is a powerful driver of motivation (see Deci, Koestner, & Ryan, 1999, for a review), and at first glance, it might seem wasteful to improve performance when rewards do not depend on performance (Lepper & Greene, 1978). However, from an optimal control perspective, both contingency and reward expectation can increase the value of performing better. This is because in environments in which the yield is likely to be higher, it is economical to capitalize on this by acting faster (Manohar et al., 2015). The increased reward can “pay the cost” of increasing speed. One consequence is that motivational vigor may be yoked to expected reward rate (Beierholm et al., 2013; Choi, Vaswani, & Shadmehr, 2014; Guitart-Masip, Beierholm, Dolan, Duzel, & Dayan, 2011; Knutson, Adams, Fong, & Hommer, 2001; Wang, Miura, & Uchida, 2013).

In the first experiment reported here, we tested whether motivational vigor was controlled by contingency or reward level in a speeded oculomotor task. Participants had to shift gaze quickly to a target after hearing a cue indicating how outcomes would be determined. To index motivational vigor, we measured the velocity of rapid eye movements (saccades), which has been shown to increase with reward (Chen, Chen, Zhou, & Mustain, 2014; Choi et al., 2014; Takikawa, Kawagoe, & Hikosaka, 2002). The cue at the start of each trial indicated whether monetary reward would be contingent on performance or guaranteed regardless of speed. A key question is whether contingent and noncontingent motivation are associated with one another. In addition to indexing motor vigor, pupil dilation provided a physiological marker of arousal in response to the motivation cues.

Penalty may also play a pivotal role in motivation (see Cameron & Pierce, 2002, for a review). We therefore asked in a third experiment whether contingent and noncontingent penalties can increase motivational vigor. To quantify this, we directly compared the effects of contingent penalties—in a condition in which participants had to perform well to avoid a penalty—and guaranteed penalties with the effects of contingent and guaranteed rewards.

## Method

In Experiment 1, we assessed increases in motivational vigor by comparing saccade velocity and pupil dilation in response to (a) performance-based rewards and random rewards and (b) guaranteed rewards and guaranteed absence of reward. Experiment 2 was identical to Experiment 1, except that performance feedback was provided only on some trials. In Experiment 3, we compared motivation by reward with motivation by penalty.

### Participants

Participants were recruited through the Oxford Psychology Research database. Participants had no neurological disorders, had normal or corrected-to-normal vision, and provided written consent approved by the Oxford University’s Central University Research Ethics Committee. In Experiment 1, 30 individuals participated (8 male, 22 female; mean age = 24.6 years, *SD* = 5.8). In Experiment 2, a further 20 participants were tested (9 male, 11 female; mean age = 24.7 years, *SD* = 4.5). In Experiment 3, 25 participants were tested (11 male, 14 female; mean age = 28.0 years, *SD* = 9.3). Sample size was determined on the basis of previous studies that used a similar incentivized saccade task with three levels of reward; these studies found motivational invigoration of saccade velocity by approximately 4% to 6%, which yielded effect sizes of approximately 0.6 to 0.8 (Manohar & Husain, 2016; Reppert, Lempert, Glimcher, & Shadmehr, 2015).

### Procedure

**Fig. 1.**
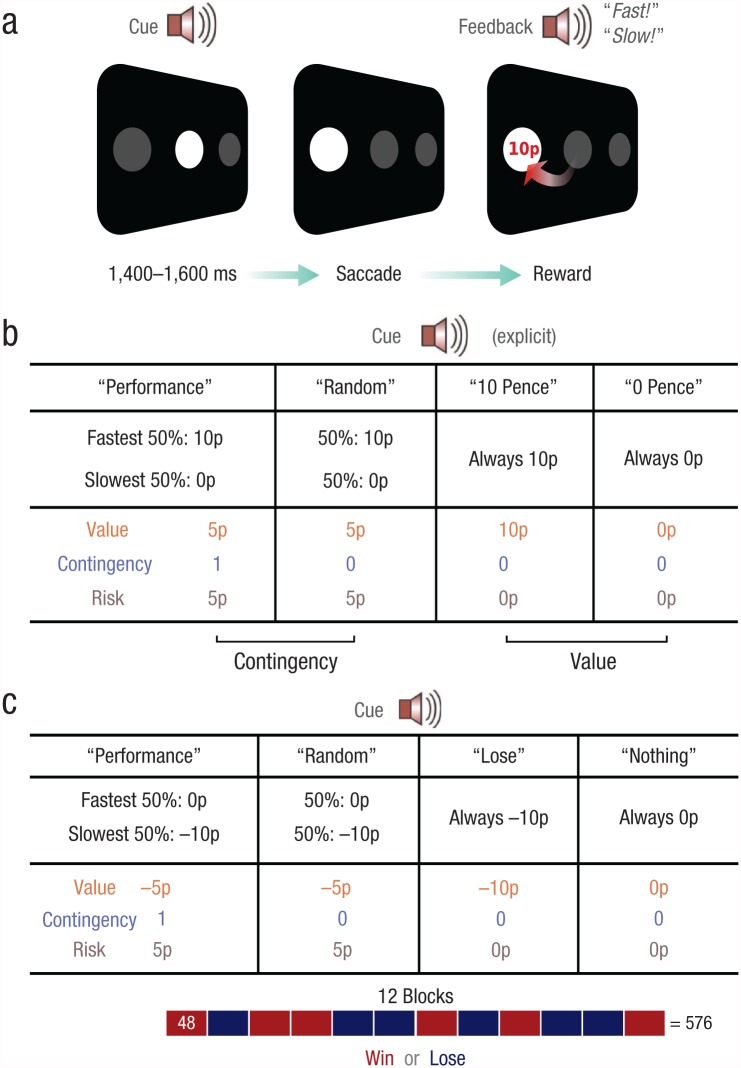
Example trial sequence and design of the experiments. On each trial (a), participants were instructed to fixate on the disc in the center of the screen. One of four cues (duration = 700 ms) informed participants how reward on that trial would be determined. A target then appeared on either the left- or right-hand side of the screen, and participants had to move their eyes to the target as quickly as possible. Once their gaze arrived at the target, participants were given feedback about their reward and also their speed on that trial. In one half of each experiment, reward feedback was visual and performance feedback auditory (as shown here); in the other half of the experiment, the modalities were switched. The chart in (b) details the four cue conditions in Experiments 1 and 2. In each column, the top row indicates the auditory cue, and the middle row shows how rewards were determined for trials with that cue. For example, in the performance-based-gains condition, the fastest 50% of reaction times were rewarded, whereas in the random-gains condition, 50% of trials were randomly rewarded. The bottom row shows the value, level of contingency (1 = fully contingent, 0 = reward unrelated to performance), and risk associated with each cue. In Experiment 3, half of the blocks were identical to those in Experiment 1, but the remaining blocks involved penalty rather than reward. The chart in (c) details the four cue conditions for the penalty blocks. These blocks mirrored those in Experiment 1, except that penalties were used in place of rewards.

In the present study, we aimed to isolate the contributions of performance contingency and reward rate on motivational vigor. We thus asked whether the mere presence of a reward itself or rather the need to act in order to obtain it controls motivational vigor. To investigate this question, we adapted the standard simple prosaccade task (Fig. 1a) by instructing participants to move their eyes as fast as possible to look at a target on either the left- or right-hand side of the screen. In Experiment 1 (Fig. 1a), we compared contingent and noncontingent motivation. On each trial, participants fixated a central disc and heard one of four spoken cues that indicated how the reward would be determined (Fig. 1b). After a variable interval (mean duration = 1,500 ms), a target disc appeared. Participants had to move their eyes to this target as quickly as possible. Once their gaze arrived at the target, participants were informed both about the time they took to reach it and about whether they received a reward.

We measured how the auditory motivational cues altered eye movement velocity. The first two conditions were used to measure contingent motivation. In the performance-based-gain condition (cue: “performance”), the fastest 50% of responses were rewarded with 10 pence, whereas in the random-gain condition (cue: “random”), 50% of all responses were randomly rewarded with 10 pence. These conditions were therefore matched for overall expected value, risk, and uncertainty but differed only in whether or not reward was contingent on performance. Therefore, any difference in movement speed between them would be solely attributable to contingency.

Motivation by noncontingent rewards was measured by comparing the 10-pence-gain condition (in which a reward was certain; cue: “10 pence”), in which reward was guaranteed, with the 0-pence-gain condition (in which the absence of a reward was certain; cue: “0 pence”), in which there was no reward. In these latter two conditions, the outcome was always certain and independent of performance but differed in expected value. Any performance differences between them could therefore be attributed only to the expectation of reward.

Participants were informed beforehand about the four cues they would hear and how the outcomes of each cue condition would be determined in each case—how quickly they reached the target (i.e., their performance), random computer selection, or award of a fixed certain amount. They performed 10 practice trials, which were followed by a debriefing to ensure they understood the conditions, and then they began the experiment. Participants completed eight blocks of 48 trials each, with all four cue types intermixed. Feedback about reward and response speed was presented on all trials: In one half of the experiment, reward feedback was visual and performance feedback auditory; in the other half, these modalities were switched (the order was counterbalanced across participants). At the end of the experiment, participants were paid according to their winnings. The experimenter was present in the room throughout the experiment.

Experiment 2 was identical to Experiment 1, except that feedback about reward and speed were presented on only 57% of trials (see Supplementary Methods in the Supplemental Material). In Experiment 3, we sought to determine whether penalties also carried similar effects as rewards on motivational vigor. The task was the same; however, half of the 12 experimental blocks (of 48 trials) used motivation by penalty instead of motivation by reward (Fig. 1c). Participants were informed at the start of each block whether it would involve rewards or penalties, and the order was randomized and counterbalanced across individuals. Penalty blocks were similar to reward blocks but had inverted reward schedules. For performance-based-loss trials, the slowest 50% of responses incurred a penalty, whereas faster responses received neither reward nor penalty. For random-loss trials, a penalty was administered on a random 50% of trials, and so this condition was matched for value and uncertainty with the performance-based-loss condition. A fixed penalty was incurred in 10-pence-loss trials, whereas no reward or penalty was given on 0-pence-loss trials. The motivation-by-reward blocks were identical to those in Experiment 1, which enabled us to directly compare motivation by reward and motivation by penalty.

### Data analysis

During the experiments, the speed of a response was indexed on-line as the time taken from cue onset until gaze stabilized within the target disc. Thus, speed feedback (and reward feedback in the conditions in which reward was contingent) was predominantly determined by saccadic reaction time. Saccades were identified using standard velocity and acceleration criteria, and the peak velocity was measured for the first saccade that landed outside the fixation disc.

We were interested in the effect of contingency (whether rewards were to be given depending on performance vs. given randomly) and the effect of reward or penalty level (10 pence vs. nothing). In each experiment, we tested this using paired-samples *t* tests to compare saccade velocity between (a) the performance-based-outcome and the random-outcome conditions and (b) the certain-outcome conditions (10-pence gain or loss) and no-reward conditions (0-pence gain or loss). Many studies have shown that incentives speed up movements (Knutson et al., 2001; Manohar et al., 2015) and consistently lead to pupillary dilation (Muhammed et al., 2016), so one-tailed *t* tests were used.

**Fig. 2.**
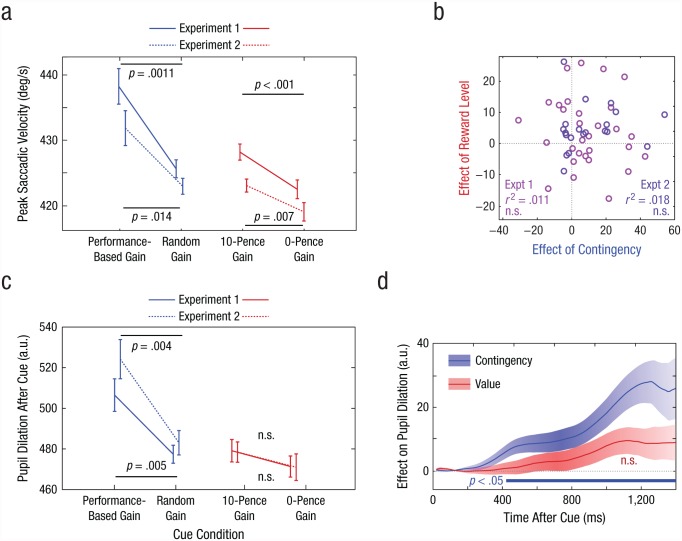
Results for measures of saccade velocity and pupil dilation in Experiments 1 and 2. Mean peak saccadic velocity (a) is shown for each cue condition, separately for each experiment. Error bars represent ±1 within-subjects *SEM*. The scatterplot (b) shows the relationship between the difference in saccade velocity in the contingency conditions (performance-based-gain and random-gain) and the reward-level conditions (10-pence-gain and 0-pence-gain). Each circle represents 1 participant. Mean pupil dilation at 1,200 to 1,400 ms after cue onset (c) is shown for each cue condition, separately for each experiment. Error bars represent ±1 within-subjects *SEM*. The mean difference in pupil dilation after the onset of the cue (d) is shown as a function of time. The blue trace shows the effect of contingency (difference between the performance-based-gain and random-gain conditions); positive values indicate that pupils were larger when performance determined the outcome than when outcomes were random. The red trace shows the effect of reward level (difference between the 10-pence-gain and 0-pence-gain conditions); positive values indicate that pupils were larger when reward was available than when it was not. Data are averaged across Experiments 1 and 2; shaded areas indicate the standard error of the difference between conditions. The blue bar indicates results that showed a significant effect of contingency (*p* < .05, as determined by a permutation test). a.u. = arbitrary units.

Pupil dilation was measured just before target onset, relative to the baseline at the onset of the auditory cue. Both the size difference and the proportional change were analyzed (see Supplementary Results in the Supplemental Material available online). To test when pupil dilation became significant after cue onset, we calculated *t* statistics using point-wise comparisons over the traces (Fig. 2d), and permutation was used to correct for multiple comparisons to maintain a family-wise error rate (α)of .05 across time points (Manohar & Husain, 2015; Nichols & Holmes, 2002). This effectively tested the null hypothesis that there would be no time points at which there was a difference between the conditions.

## Results

### Experiment 1

In Experiment 1, peak saccade velocity was significantly greater when rewards were performance-based (*M* = 438°/s^–1^, *SEM* = 11.1) than when they were random (*M* = 426°/s^–1^, *SEM* = 11.1), *t*(29) = 3.38, *p* = .0011, which indicates that movement was invigorated by contingency (i.e., when reward was performance dependent; Fig. 2a). This finding is consistent with performance-related rewards being one of the strongest drivers of motivation (see Ryan, Mims, & Koestner, 1983, for a review). But peak saccade velocity was also significantly greater in the 10-pence-gain condition (*M* = 428°/s^–1^, *SEM* = 11.0) than in the 0-pence-gain condition (*M* = 422°/s^–1^, *SEM* = 10.5), *t*(29) = 3.58, *p* = .001, which indicates that reward expectation increased vigor independently of contingency. Thus, even when rewards could be obtained unconditionally, they still increased motivational vigor.

Are people who are motivated by contingency also motivated by the mere expectation of certain reward? To address this question, we examined the correlation across individuals between two effects: contingency and reward level. These effects were measured by taking the difference between each participant’s saccade velocity in (a) the performance-based-gain and random-gain conditions (effect of contingency) and (b) the 10-pence-gain and 0-pence-gain conditions (effect of reward level). These effects were not significantly correlated across individuals (Fig. 2b), which suggests that there was no relationship between the two kinds of motivation (Pearson’s *r* = .10, *p* > .25).

A hallmark of true motivational improvements is that increases in speed are not accompanied by reductions in accuracy (as opposed to trading speed for accuracy; Manohar et al., 2015). Variability in movement end points, calculated as the standard deviation of saccade amplitude for each condition, did not increase in the performance-based-gain condition (*M* = 1.09°) relative to the random-gain condition (*M* = 1.16°), *t*(29) = 1.07, *p* = .15, and was in fact reduced in the 10-pence-gain condition (*M* = 1.07°) relative to the 0-pence-gain condition (*M* = 1.29°), *t*(29) = 2.44, *p* = .011. In addition to variability, the bias toward making smaller saccades (*hypometria*, or undershoot) was also smaller for the 10-pence-gain condition than the 0-pence-gain condition, which indicates that reward reduced error (see Supplementary Results). Velocity increases exceeded those expected from the amplitude increase with motivation (see Fig. S7 in the Supplemental Material). Thus, increases in velocity did not come at the expense of accuracy.

Previous studies demonstrating pupillary dilation in response to incentives used only performance-dependent rewards, such that both reward level and contingency varied together (Chiew & Braver, 2013; Knutson et al., 2001). Moreover, risk and uncertainty about the outcome also increased with higher incentives, both of which can result in pupillary dilation (Satterthwaite et al., 2007). A key question, then, is to what extent autonomic arousal is sensitive to performance contingency or sensitive to reward expectation per se.

Pupil dilation between cue onset and target onset was significantly greater in the performance-based-gain condition, when rewards were contingent on speed (change in size: *M* = 398 units, *SEM* = 35), than when rewards were given at random (change in size: *M* = 374 units, *SEM* = 32; Fig. 2c), *t*(29) = 2.73, *p* = .0054. However, pupil dilation was not significantly different in the 10-pence-gain condition (*M* = 376 units, *SEM* = 32) than in the 0-pence-gain condition (*M* = 369 units, *SEM* = 31), *t*(29) = 0.99, *p* = .17. These findings demonstrate that autonomic arousal is clearly increased when reward depends on performance, even when uncertainty is controlled for. On the other hand, simply anticipating a guaranteed reward increased arousal only marginally. One possible interpretation of this dissociation is that the pupillary dilation observed when incentives were available could relate to increased effortful engagement in order to obtain rewards (Beatty, 1982) rather than simply reflecting purely motor invigoration that occurs even with unconditional rewards.

The findings of Experiment 1 are therefore in keeping with two separable and independent motivational effects. Consistent with our hypotheses, results showed that contingency led to increased motivational vigor, as measured by peak saccadic velocity. This motivational effect of contingency was distinct from a second effect, in which increased reward expectation also increased motivational vigor. Changes in pupil dilation suggest that performance contingency is a strong driver of autonomic arousal, unlike reward rate per se.

### Experiment 2

Experiment 2 replicated these findings on a separate group of 20 healthy volunteers (Figs. 2a and 2c). (This experiment was identical to Experiment 1, except that feedback was probabilistic.) There were significant invigorating effects of both contingent rewards, *t*(19) = 2.69, *p* = .007, and noncontingent rewards, *t*(19) = 3.00, *p* = .004. There was no correlation between these two effects, which supports the dissociation observed in Experiment 1 (*r* = .13, *p* > .25; Fig. 2b). Contingency increased pupillary dilation, *t*(19) = 4.46, *p* < .001, but reward level did not, *t*(19) = 0.44, *p* = .25. Performance was slower overall than in Experiment 1, perhaps because the reduced feedback lowered the global motivational state. In both Experiments 1 and 2, we also investigated whether a history of rewards on previous trials had any effects, but none were observed (see Fig. S2 in the Supplemental Material).

To investigate the time course of arousal, we analyzed the entire pupil trace after the cue up to 1,400 ms in both Experiments 1 and 2 (Fig. 2d). The change in pupil size between the performance-based-gain condition and the random-gain condition became significantly different at 400 ms and remained significant until after the target appeared. Pupils did not significantly dilate more when rewards were available than when no rewards were available.

### Experiment 3

**Fig. 3.**
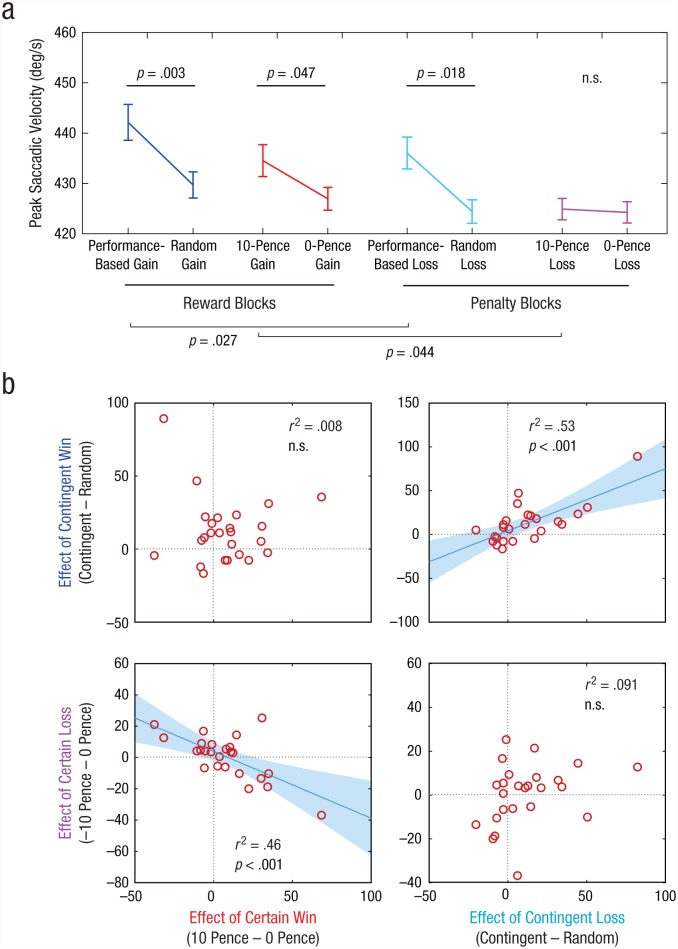
Saccade-velocity results for Experiment 3. Mean peak saccade velocity (a) is shown as a function of cue condition, separately for reward and penalty blocks. Error bars represent ±1 within-subjects *SEM*. The four scatterplots in (b) show the correlation between the differences in saccade velocity for contingency in reward blocks and reward level in reward blocks (top left), contingency in reward blocks and contingency in penalty blocks (top right), reward level in penalty blocks and reward level in reward blocks (bottom left), and reward level in penalty blocks and contingency in penalty blocks (bottom right). Best-fitting regression lines are shown for the graphs in the bottom left and top right.

In the performance-based-loss trials in Experiment 3, when penalties were contingent on fast performance (*M* = 437°/s^–1^, *SEM* = 14), saccade velocity was faster than in the matched random-loss condition (*M* = 427°/s^–1^, *SEM* = 12), *t*(24) = 2.22, *p* = .018 (Fig. 3a). This indicates that the prospect of a penalty could, just like the prospect of a reward, induce motivational benefits when it depended on performance. However, velocity was not increased or decreased in the 10-pence-loss condition (*M* = 427°/s^–1^, *SEM* = 12) compared with the 0-pence-loss condition (*M* = 434°/s^–1^, *SEM* = 12), *t*(24) = 0.79, *p* = .21. This finding is in contrast with the effect of certain reward. The reward blocks in Experiment 3 provided a replication of Experiment 1, demonstrating invigoration by performance-based rewards (*M* = 443°/s^–1^, *SEM* = 45), compared with random rewards (*M* = 430°/s^–1^, *SEM* = 14), *t*(24) = 2.96, *p* = .003, and by certain rewards (*M* = 435°/s^–1^, *SEM* = 14), compared with no rewards (*M* = 427°/s^–1^, *SEM* = 13), *t*(24) = 1.74, *p* = .047.

A 2 (value: 0 pence vs. 10 pence) × 2 (block: reward vs. penalty) analysis of variance (ANOVA) yielded a main effect of block, *F*(1, 24) = 4.96, *p* = .036, demonstrating that expecting a reward led to greater effects of value than expecting a penalty (there was no interaction between value and block). The effect of block was driven by slower responses to fixed penalties than to fixed rewards—pairwise post hoc *t*(24) = 2.02, *p* = .027; there was no difference between reward and penalty blocks for the 0-pence conditions, *t*(24) = 0.79, *p* = .22. However, a 2 (contingency: performance-dependent outcome vs. random outcome) × 2 (block: reward vs. penalty) ANOVA revealed that there was no difference between contingent reward and contingent penalty, *F*(1, 24) = 2.62, *p* = .25. This suggests that the prospect of an unavoidable penalty did not result in faster or slower movement and was thus not simply the opposite of certain reward.

To establish whether motivation by penalty is qualitatively distinct from motivation by reward, we examined between-subjects correlations in the size of motivational effects. Four distinct effects were measured for each individual: contingent reward, contingent penalty, certain reward, and certain penalty, each measured relative to their respective control conditions (Fig. 3b). We examined four correlations to discover whether different people respond more to reward or to penalty or whether different people respond to contingent or noncontingent motivation.

The dissociation observed in the first two experiments was replicated in a new sample: contingent and noncontingent motivation again did not correlate across individuals. This was true for both reward (*r* = .089, *p* > .25) and penalty (*r* = .30, *p* = .14). But the responses to contingent reward and contingent penalty were highly correlated (*r* = .73, *p* < .001), as were responses to certain reward and certain penalty (*r* = –.68, *p* < .001). This indicates first that people whose performance improved when offered a reward dependent on their performance also improved when a penalty depended on performance. Second, an orthogonal trait is the tendency to improve performance for guaranteed rewards—and the same individuals tended to reduce their movement speed for unavoidable penalties. Note that at a group level, certain penalties had no net effect on movement speed, but there was variability among individuals (i.e., some individuals slowed down and others sped up). This variability in the penalty effect is strongly related to the increase in speed with certain rewards. In other words, guaranteed penalties did influence movement speed, but unlike guaranteed rewards, which always tend to increase speed, penalties increased or decreased movement speed in different people. Thus, contingency and reward level determined motivational vigor in a consistent way for reward and penalty, but they operated independently of one another.

**Fig. 4.**
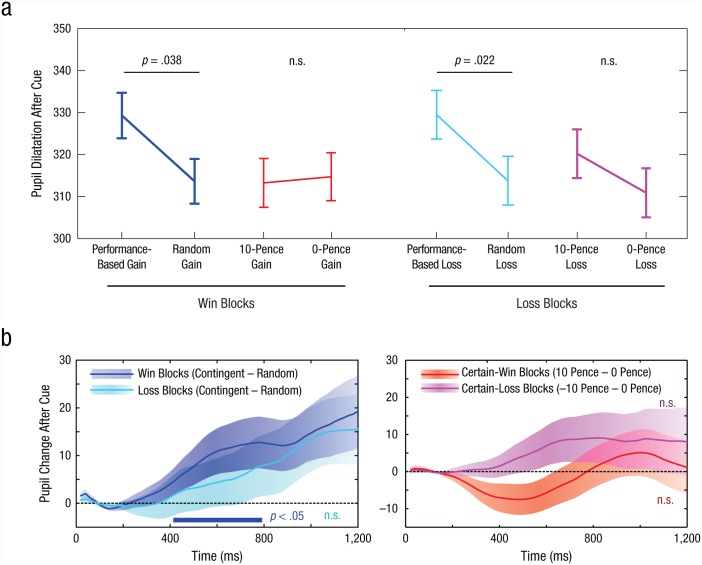
Pupil-dilation results in Experiment 3. Mean pupil dilation 1,200 to 1,400 ms after the onset of the cue (a) is shown as a function of cue condition, separately for reward and penalty blocks. Error bars represent ±1 within-subjects *SEM*. The graphs in (b) show the mean difference in pupil dilation after the onset of the cue as a function of time. In the left graph, the darker blue trace shows the effect of contingency (difference between the performance-based-gain and random-gain conditions) in the reward blocks, and the lighter blue trace shows the effect of contingency (difference between the performance-based-loss and random-loss conditions) in the penalty blocks; positive values indicate that pupils were larger when performance determined the outcome than when outcomes were random. In the right graph, the darker red trace shows the effect of reward level (difference between the 10-pence-gain and 0-pence-gain conditions) in the reward blocks, and the lighter red trace shows the effect of contingency (difference between the 10-pence-loss and 0-pence-loss conditions) in the penalty blocks; positive values indicate pupils were larger when reward was available than when it was not. Shaded areas indicate the standard error of the difference between conditions. The blue bar in the left graph indicates results that showed a significant effect of contingency (*p* < .05, as determined by a permutation test).

Do penalties also generate autonomic arousal? Pupil diameter increased in response to performance-based penalties, compared with random penalties, *t*(24) = 1.83, *p* = .040 (Fig. 4). However, no effect of arousal was found for unconditional penalties, *t*(24) = 1.16, *p* = .13. This lack of arousal echoes what was observed for unconditional rewards. In reward blocks, the results of Experiment 1 were replicated, showing pupillary dilation for contingent rewards, *t*(24) = 2.46, *p* = .011, but not for noncontingent rewards, *t*(24) = 0.09, *p* > .25.

Reaction times showed small and inconsistent speeding with motivation, but these effects did not explain velocity increases **(**see Supplementary Materials and Fig. S5 in the Supplemental Material).

## Discussion

In these experiments, we aimed to compare the effects of performance-contingent incentives with the effects of noncontingent rewards. Results across the experiments established that the impact of incentives on motivational vigor (e.g., Beierholm et al., 2013; Knutson et al., 2001) can in fact be divided into two distinct effects: the effect of contingency and the effect of reward expectation. Movement velocity increased and pupils dilated when performance-contingent rewards were available, relative to when random rewards were available, and this was the case in all experiments (Figs. 2 and 4). Expected reward level also had motivational effects distinct from effects of contingency, because a fixed high reward resulted in faster movements than no reward. Although both these aspects drove motivational vigor, they varied independently of one another across individuals. Arousal was primarily driven by contingent incentives. Experiment 2 replicated Experiment 1 under conditions of greater uncertainty, and Experiment 3 demonstrated that contingent penalties generated motivation, whereas unavoidable penalties invigorated or retarded movement in different individuals (Fig. 3).

Why are there separate mechanisms for contingency- and reward-related motivation? Unlike reward-driven motivational vigor, contingency-driven motivation might specifically reflect goal-directed aspects of instrumental behavior or may require an internal model of causality in the world (Dayan & Berridge, 2014). According to this view, invigoration by reward reflects model-free optimization of engagement, whereas contingency may promote model-based engagement (Daw & Dayan, 2014). Similar dissociations have been demonstrated in higher cognitive mechanisms, such as cognitive control (Braem et al., 2013; Braem, Verguts, Roggeman, & Notebaert, 2012; Chiew & Braver, 2013, 2014; Fröber & Dreisbach, 2016; Stürmer, Nigbur, Schacht, & Sommer, 2011; van Steenbergen, Band, & Hommel, 2009).

Why should reward speed up movement if it can be obtained unconditionally? One attractive explanation is that time carries an opportunity cost that must simultaneously be weighed against the increased effort needed for faster movements (Harris & Wolpert, 2006; Manohar et al., 2015). The opportunity cost of time is amplified when reward is expected (Kacelnik, 1997)—a mechanism which may depend on tonic dopamine (Niv et al., 2006). Curiously, robust reaction time (RT) effects were not observed in the present experiments, unlike in other studies (Guitart-Masip et al., 2011). Exogenous prosaccades, a highly reflexive brain-stem response, may be somewhat immune to RT modulation (Kveraga, Boucher, & Hughes, 2002), whereas motivational vigor or velocity is determined at this primitive level.

We crucially controlled for the level of intrinsic motivation across conditions (Cerasoli, Nicklin, & Ford, 2014). One key determinant of intrinsic motivation is performance feedback (Harackiewicz, 1979; Pittman, Davey, Alafat, Wetherill, & Kramer, 1980), which we equated across conditions. Moreover, unlike in historical studies, trials were interleaved, so that key motivating variables such as performance expectations and time on task were matched across our conditions of interest. Guaranteed rewards were still task dependent and thus extrinsic (Ryan et al., 1983). Intrinsic motivation could still influence utility differences between reward levels, although explicit monetary cues mitigate this (Deci et al., 1999). Verbal reward cues minimized the need for learning in comprehending the cue. Instead, long-term associations could have generated task-independent, non-goal-directed motivational vigor (Niv, 2007).

The present study also disentangles contingency effects from the accompanying uncertainty, which can dilate the pupil (Satterthwaite et al., 2007), alter subjective value (Tversky & Kahneman, 1981), or even boost motivation (Shen, Fishbach, & Hsee, 2015).

## Conclusion

Across three experiments employing a simple measure of motivational vigor, this study isolated two independent contributions to motivation. The phenomenon of invigoration by incentives reported in previous studies is partly due to performance contingency and partly due to reward expectation itself. Here, contingency was shown to increase motivational vigor both for rewards and penalties, whereas certain rewards but not certain penalties were motivating. Taken together, these findings imply that invigoration by incentives can be largely attributable to the two fundamentally separate effects of contingency and reward rate.
